# Soft Drinks and Sugar-Sweetened Beverages Advertising in Spain: Correlation between Nutritional Values and Advertising Discursive Strategies

**DOI:** 10.3390/ijerph17072335

**Published:** 2020-03-30

**Authors:** Mireia Montaña Blasco, Mònika Jiménez-Morales

**Affiliations:** 1 Faculty of Information and Communication Sciences, Universitat Oberta de Catalunya, 08035 Barcelona, Spain; 2 Department of Communication, Pompeu Fabra University, 08018 Barcelona, Spain; monika.jimenez@upf.edu; 3 UPF Barcelona School of Management, 08008 Barcelona, Spain

**Keywords:** childhood obesity, food advertising, sugar-sweetened beverages, soft drinks, food policy, nutrient profiling

## Abstract

Spain ranks fifth among European countries for childhood obesity. Sugar-sweetened beverages (SSBs) and soft drinks (SDs) are consumed by 81% of the Spanish children weekly. Advertising is one of the factors that contributes to an obesogenic environment. This study correlated longitudinally the nutritional values of SSBs and SDs and advertising discursive strategies between 2013 and 2018 for all media. A mixed-methods approach was applied that included a quantitative analysis of advertising spend data, a content analysis and a study of the discursive strategies used in advertisements. In addition, the Nutri-score system was used in order to determine the nutritional quality of the beverages. The results were analyzed applying the Spanish advertising regulatory framework for obesity prevention. The main findings indicate an association between low nutritional value beverage advertisements and a discourse based on hedonistic elements. In order to prevent childhood obesity in Spain, a stricter regulation of advertising is necessary, especially in aspects such as the language used to present products and celebrity endorsements.

## 1. Introduction

One in three Spanish children is obese or overweight. This condition is a serious problem for public health, not only because of the extra medical cost of millions of euros [1], but also because of its impact on physical and psychological factors among minors [2,3]. Advertising is one of the factors that greatly favors the obesogenic environment: Spanish children are exposed to an average of 9000 TV advertising campaigns per year [4], and the vast majority of these advertisements are for products of little or no nutritional value [5].

Among the low or non-existent nutritional value of most of the advertised products in Spain, soft drinks (SDs) and sugar-sweetened beverages (SSBs) are highlighted. It is estimated that the consumption of SDs by families with children under the age of 14 was almost 70 L per family in 2018. The consumption of SSBs in the same group was almost 16 L per family unit. In this sense, scientific data indicate that Spanish children under nine years consume soft drinks and sugary drinks on a regular basis: 7% consume SDs and SSBs daily, 16% almost every day (4–6 days), 58% some days (1–3 days) and only 19% consume these types of drinks less than once a week. Also, 12% of this consumption takes places during school breakfast [6].

Several studies have indicated a relationship between the consumption of sugar-sweetened beverages and soft drinks and childhood obesity [7,8,9,10,11,12,13,14]. Some of them have also emphasized that sugar-sweetened beverages are the primary source of added sugar in the diets of children [8,15]. The scientific literature coincides in stating that among the policies necessary to reduce the consumption of SSBs and SD among children, there should be a strict review of the advertising of beverages aimed at that group [16,17,18,19,20,21,22,23,24,25,26,27,28,29]. 

With the aim of reducing the continuous growth of childhood overweight, the Spanish Agency for Consumer Affairs, Food Safety and Nutrition (AECOSAN, Agencia Española de Consumo, Seguridad Alimentaria y Nutrición) in 2005 launched what is known as the NAOS Strategy (Strategy for Nutrition, Physical Activity, and the Prevention of Obesity) [30]. Among its priorities, the NAOS strategy intends to reduce the impact of food marketing on children through a self-regulating code of advertising. Part of this strategy is conveyed to the PAOS Code (Code of Co-regulation of Advertising for Food Products and Beverages directed to Children, Prevention of Obesity and Health) [31], which, together with the Spanish Federation of Food and Beverage Industries, aims to co-regulate advertising messages targeted at children under the age of 12. It should be noted that the PAOS Code is especially focused on television advertising and does not take into account other types of advertising media. The only exception is the Internet, in that the Code establishes a regulation in advertising aimed at children under 15 years of age. Part of the PAOS Code refers to the language used for the presentation of the product, the benefits obtained and the use of celebrity endorsements that could be an element of influence during a purchase. 

Several studies agree in affirming the inefficiency of the PAOS Code. In this sense, Royo Bordonada et al. [1] have stated that Spain, like other European countries, has opted for educational measures of insufficient efficacy by focusing on individuals. These measures have been unable to reverse the obesity epidemic. According to the authors, the NAOS Strategy is based on “educational measures and in doubtful public-private partnerships, embodied in voluntary and self-regulation agreements”. As such, Romero-Fernández et al. [32] noted that almost 50% of ads from companies in the food and beverage industry that have signed up to the PAOS Code fail to comply with its tenets. 

Scientific research carried out in Spain so far has focused on the study of how advertising presents low nutritional value foods to children, and how that presentation violates the PAOS Code. Images, speech and the use of licensed or famous characters [4,5,20,25,32,33] are some of the analyzed aspects in relation to food advertising aimed at children. These studies, however, have focused specifically on television advertising, and have not taken other media into consideration. In addition, scientific literature in Spain does not take into account the advertising of SSBs or SDs either, since it focuses only on food.

The purpose of this study is to find associations between SSB and SD advertisements across different types of media (TV, print, radio, outdoor/Out-of-Home (OOH) and the Internet), the language used and the low nutritional value of products. To that end, we selected the campaigns made by the top 10 companies of these categories between 2013 and 2018.

The results obtained are contrasted with the guidelines determined in the regulatory framework of the PAOS Code in order to examine whether they have been effective. The analysis pays special attention to determining whether advertisers who explicitly express their adherence to the PAOS Code apply the regulations to the advertising discourse they use.

## 2. Materials and Methods 

### 2.1. Material Design and Procedure

This cross-sectional study was designed based on a mixed-methods approach. A quantitative methodology was used to compile spending data on SD and SSB advertising. These data were extracted from Infoadex, the company that monitors advertising activity in Spain, during the period between 2013 and 2018. This company has the largest database of the sector, classified with the highest level of detail and with quantitative data (insertions, spending and occupation) and qualitative data (advertisements). 

We took into consideration all types of media that Infoadex monitors: television, press, magazines, radio, outdoor and internet. In this sense, a qualitative analysis was carried out based on the main SD and SSB advertising campaigns that were captured by the Infoadex Mosaico. To that end, we selected the campaigns made by the top 10 companies of these categories (a total of 78 products). In total, we considered a total of 4956 advertisements in order to perform a content analysis based on the following proportion: radio 29%, internet 26%, television 22%, press 10%, magazines 8%, outdoor 4%, and cinema 1%.

### 2.2. Nutritional Analysis

To perform this analysis, we classified the different products advertised by considering the following variables: kind of beverage (SSB or SD), and nutritional value of each product. Once the kind of beverage was determined, the nutritional value was estimated using the Nutri-score, a system for assessing the quality of foods and beverages. Nutri-score classifies products into colors from red to green, depending on their nutritional values. Red is for low nutritional value products, orange is for medium nutritional value foods and beverages, while green is for products of high nutritional value.

Several studies have validated the Nutri-score system’s effectiveness by analyzing its applications in different countries like France and, recently, Belgium, Germany and Portugal, which are in the process of introducing it [34,35,36,37,38,39,40].

This nutritional analysis focused on SSBs and SDs advertised in Spain, a country that approved the application of the Nutri-score system in 2018. Almost two years later, adoption of the nutritional trac light on labels by manufacturers is on a voluntary basis. At the moment, it is not mandatory to include the color resulting from the nutritional analysis of the Nutri-score system in food and beverage labels. For this reason, the majority of products examined did not have a nutritional label. For the nutritional analysis, we applied the Nutri-score system to all of the SSBs and SDs that appeared in ads between 2013 and 2018.

### 2.3. Language Analysis

In order to analyze the language used in SSB and SD ads, we considered the transcription of the 4.956 creative items advertised between 2013 and 2018. Next, we analyzed the lexical units that were used in the discourse content. We understood the concept “lexical unit” as a “word taken in one well-specified sense and supplied with all the information specifying its behavior when it is used in this sense” [41]. 

We identified the three lexical units most used by each advertised product and classified them into semantic fields, grouping them into sets that shared a common core of meaningful characteristics. For this, we relied on the classic definition of the semantic field concept: “a group of words that stand in paradigmatic opposition to one another and share at least one semantic component” [42]. The whole content analysis process was coded by the authors using Excel. An external researcher was requested to check the intercoder reliability.

The analysis of lexical units and their groupings into semantic fields allowed us to determine the type of discourse used in the presentation of the SSB and SD ads. We also analyzed the relationship between the description of the product and the nutritional characteristics of the beverages.

The classification into semantic fields was specifically adapted to the typology of lexical units identified in each of the selected advertisements. To achieve the study objectives, the following variables were considered in the analysis of the lexical units: product, nutritional value, semantic field and lexical units used in the advertisement.

### 2.4. Sponsorship and Celebrity Endorsement Analysis

For this point, we analyzed the use of sponsorship and celebrity endorsement on the advertisements. Based on this, we determined three categories of sponsorship or celebrity endorsement, as follows: sports organizations, musicians and TV hosts.

### 2.5. PAOS Code Analysis

In spite of the fact that SSB and SD advertisements not only target children, it should be noted that children are one of their main consumers in Spain [6]. For this reason, we related the content of the ads with the regulatory framework of the PAOS Code. For this purpose, we focused the study on point 5 of section IV of the code, which refers to the product presentation; point 9 of section V, which refers to the language used in advertising aimed at minors; point 12 of section VI, relative to the benefits of the product; and point 14.1 of section VII, which focuses on the endorsement and promotion of the product through characters and programs.

## 3. Results

### 3.1. Spending and Nutritional Analysis

We must highlight an annual increase in advertising spending on SD and SSB since 2014, which increased from nearly 32 million Euros in 2013 to over 53 million Euros in 2018. A total of 95.7% of the advertising spending in the category of SSBs and SDs is made by the top 10 companies leading the market in Spain.

The results revealed that of the advertising spending from the analyzed products, only EUR 812,061 (0.3%) corresponded to products with a high nutritional value (termed an “A” beverage nutritional label). A total of 8.7% of beverages had a medium–high values (B label), and 10.4% had a medium nutritional value (C label), while the nutritional value of 17.9% of the advertised beverages was low (D label), and 62.7% was very low (E label).

Data from the analyzed years found that 2015 was the period with the highest spending on D and E label products (91.8%). This spending has been falling proportionately since then, standing at 74.0% in 2018 (Table 1). This fact is mainly due to the launch of new varieties of products with less added sugar (e.g., Fanta Zero, Sprite Light, Trina Zero).

The results of advertising spending by category revealed that 79% of soft drinks and 90% of sugar-sweetened beverages corresponded to low (D label) or very low (E label) nutritional values (Table 2). 

Taking into consideration the spending data by media, results showed that nearly 72% of the spending was in television (71.9%), followed by outdoor (15.0%) and cinema (3.7%), although with a much lower contribution.

The spending on products labeled D and E was most prominent in all of the analyzed media, especially in radio (86.9%), outdoor (86.5%) and cinema (86.5%) (Table 3).

### 3.2. Language Analysis

We carried out a linguistic analysis based on the message transmitted in the discourse of the ad in order to determine the use of language in relation to the nutritional value of the products. 

To this end, we analyzed the three most used lexical units for each product (234 in total) and classified them into a set of semantic fields, which showed a common core of meaningful features between these lexical units. The intercoder reliability coefficient was 0.91. We established a correlation between the nutritional value and the semantic field that described the product.

It is noteworthy that the most used semantic fields referred to the qualities of the advertised beverages (e.g., sabor (taste), burbujas (bubbles, presence of carbon dioxide gas (CO_2_)), sin azúcar or light (sugar-free), auténtico (authentic), ecológico (ecological)). A total of 126 (54%) of the analyzed words belonged to this category.

The second most used category was moods, derived from the consumption of the product (e.g., disfruta (enjoy), sé único (be unique), sentirte bien (feeling well), felicidad (happiness)), representing 13% of the total.

Action was the third category of semantic fields, with 12% of the words analyzed (e.g., Despierta tus sentidos (wake up your senses), dar un giro (take a turn), retornar (come back), ser curioso (be curious), romper con lo establecido (break with the established)).

Other semantic fields used less frequently were those related to leisure time (9%), beverages (7%), meals (3%) and price (1%) (Table 4).

### 3.3. Sponsorship and Celebrity Endorsement Analysis

With respect to the use of sponsorship and celebrity endorsements in the ads analyzed, we found that this kind of strategy was regularly used as a part of the advertising strategy to attract consumers’ attention. The association with sports organizations was one of the tools most commonly used. During the period studied, we found five products associated with ADO (Olympic Sports Association) (33 advertisements), one with the UEFA Champions League (10 advertisements), one with UEFA Euro 2016 France (1 advertisement) and one with Universo Mujer Baloncesto (Universe Woman Basketball) (1 advertisement). These advertisements were associated to four products with E nutritional labels, one with a D label, two with C labels and one with a B label.

Celebrities were also used as a part of the advertising strategies. We found 62 advertisements related to this category. One product labeled as E used a Spanish band, and nine products used TV program hosts. These products were labeled as follows: four were E, one was D, one was C and three were B.

### 3.4. PAOS Code Analysis

Some of the analyzed expressions such as “be unique” (as in being special in some way) or “happiness” (as in feeling or showing total pleasure or satisfaction) associated with drinking a certain beverage could be confusing for vulnerable targets, including children. Such expressions can make these targets believe that the consumption of the product can have a beneficial effect on their lives and on their social relationships. 

Taking into account that these benefits are not directly derived from the use of the product, it should be noted that the expressions used do not conform to point 5 of section IV of the PAOS Code. This point states that advertisements of food products or beverages disseminated in audiovisual and printed media shall not mislead children with regards to the benefits derived from the use of the product. In this sense, the PAOS Code also clearly states that the advertisement must not give the impression that acquiring or consuming food products or beverages will make the child more acceptable among his/her friends or suggest that the product provides the user with prestige, abilities or other special qualities.

Furthermore, the analysis showed lexical units denoting aspects that were not inherent to the product. “Fun” or “surprising” are some examples of expressions that do not comply with the regulations of the PAOS Code. In this sense, point 12 of section VI sets out that in food and beverage advertisements in audiovisual and printed media aimed at children, the benefits attributed to the food product or beverage must be inherent to its use. In no case could the aforementioned expressions be considered attributes inherent to their products.

At present, 47 food and beverages companies support the PAOS Code, thus showing their commitment to respecting all the points it comprises. The total number of companies that have signed up to the code accounts for almost 80% of advertisers in the food and beverage industry.

## 4. Discussion

According to data from the Spanish Media Research Association (AIMC, Asociación de Investigación en Medios de Comunicación), 99% of children watch TV, and 80% do so daily. The highest levels of TV viewing by minors occurs outside the legal time slot. An average audience of 19% of this group was recorded—more than 1.2 million minor spectators—between 10:00 p.m. and 12:00 a.m. (prime time), with 5.4% of children also remained in front of the TV after midnight [4,33].

As far as we know, this is the first study to analyze SSB and SD advertising content in all media in Spain. Results from this study show that 81% of the advertising spending for SSBs and SDs in Spain goes to products of low (D) or very low nutritional quality (E). Despite the companies responsible for these SSBs and SDs launching healthier versions of classic drinks (e.g., light, zero, etc.) to the market (according to the Nutri-score system), the truth is that spending continues to focus on those with the lowest nutritional values. 

Although all of the companies that invested in advertising of SSBs and SDs expressed their adherence to the regulatory framework established by the PAOS Code to prevent childhood obesity, the study indicates that companies’ advertising goals may be at odds with children’s health. 

This idea coincides with the results of previous research that showed the ineffectiveness of the self-regulatory measures of the PAOS Code as a tool to prevent childhood obesity in Spain [1,4,5,20,25,32,33]. However, the aforementioned studies did not analyze other advertising media beyond TV or the advertising language used to attract children’s attention.

The language analysis indicates that the association between moods and beverages may have adverse consequences among vulnerable targets. Adventure, pleasure, friendship, happiness, success and fun were some of the concepts originating from the language that appeared in the advertisements showing products with a low or a very low nutritional value. 

In this sense, the language that was used in the ads had connotations of prestige and the gaining of certain abilities or greater social acceptance. The constant allusion to aspects like happiness, strength, power and victory were usually highlighted as a benefit of the beverage in most of the advertisements. 

Exposure to SSB and SD advertising has been related to increased consumption of those products by children and adolescents. According to Battram et al. [19], advertising plays an important role in choosing a drink for the first time. Indeed, the “taste” concept constantly appears in ads as a product benefit; in fact, taste and preference ultimately appear to determine a child’s choice of beverage over time. 

Concerning this, age is a determining factor: at 12 years of age, children have the cognitive skills to evaluate advertising from a critical point of view. However, it is not until late adolescence or early adulthood that people really understand the persuasive nature of advertising. Teenagers primarily focus on the perceived images and role of a beverage in social functioning (i.e., conformity with peer group norms). On the other hand, children are more focused on the novelty of a product, as well as the feeling of entertainment that an advertisement provides [9,18,43,44]. In this sense, the current research found that the use of expressions such as “premium”, “fun”, “enjoy” or “positive” were commonly used to attract consumers. 

The language used in the Spanish advertisements of SSBs and SDs with low nutritional value in no way helped prevent obesity among children. In fact, the opposite way true: they promoted good experiences and positive emotions as a purchase benefit. Scientific literature concludes that introducing hedonic elements into food and beverage advertising can affect sensitive targets like children, causing them to experience phenomena related to the addiction of sensitization by incentives or disorders such as bingeing [45].

The language used by SSBs and SDs does not take into consideration point 12 section VI of the PAOS Code. Following the Code, the benefits that advertising ascribe to the beverage must be inherent to it. The advertisements must not suggest to children that the consumption of a product can provide special qualities, prestige or popularity among his/her social environment.

The PAOS Code establishes the regulatory framework for the advertising design of foods and beverages aimed at children. In order to be totally effective at obesity prevention, this regulation should be extended to those unhealthy products that children consume in spite of not being specifically targeted to them.

Nevertheless, this self-regulation does not take into consideration that language is used to draw attention to qualities extrinsic to products, offering a positive emotional reward as a consumption benefit to children. According to several studies, this reward may be a highly influential factor in the buying decision process [24,25,26].

For this reason, concepts such as “be strong”, “be happy” or “learn”, among others, can in no way be understood as benefits inherent to consumption of the product. The same thing happens with the use of these lexical units that suggest social recognition or acceptance by consuming the beverage.

Regarding celebrities and popular characters, the PAOS Code suggests they should not appear, taking into consideration that they may influence the consumption of the product. Point 14.1 of section VII points out that celebrities or popular characters should not appear in food and beverage advertisements aimed at minors. Given the fact that this study analyzes SSB and SD advertising in different media and that, except for the legal time slot for the protection of minors on television, these media do not present restrictions for children, the recommendations of the PAOS code in this regard are not effective.

In this sense, children not only receive the impact of advertising aimed specifically at them, but additionally are the perfect target for any type of advertising stimulus that appeals in an attractive way to them.

Even though paternalistic government restrictions on food and beverage advertising may be questioned, the existing regulatory framework should be reviewed [28,33]. Voluntary regulatory codes from many countries (e.g., United States, United Kingdom, Australia, New Zealand) have been ineffective in reducing exposures to non-healthy food advertising aimed at children [29,46,47,48]. On the contrary, stricter restrictions (e.g., Quebec, Norway, Sweden) confirm that a ban on this kind of advertising can be effective in lowering non-healthy food consumption and increasing social welfare [33,49,50].

As a regulatory measure, some countries have increased taxes on SSBs and SDs. In California, the consumption of taxed beverages fell by 21% in the four months after the tax was implemented [51]. After one year of its introduction, the Catalonian excise tax on SSBs brought about a 39% fall in the prevalence of regular consumers of taxed beverages [52]. Also, SSB purchases decreased and water purchases increased after an SSB tax was imposed in Mexico. The magnitude of these changes was greater in lower-income and urban households [53].

This research shows the importance of highlighting the need for stricter regulations in beverage advertising. At the same time, the results also show the need to take, as an example, some other countries that have developed tools to evaluate marketing and advertising policies within and across geopolitical boundaries to protect children’s diets and health [7]. In order to prevent a health problem with implications as serious at various levels as childhood obesity, advertising strategies that do not comply with the regulations should be sanctioned. 

This study has many strengths, and some limitations as well. To our knowledge, this research is the first to longitudinally correlate some of the variables involved in childhood obesity prevention strategies implemented in Spain, the nutritional quality of SSBs and SDs, advertising spending, as well as the advertising strategies used to attract the attention of consumers in all media. 

One of the most remarkable strengths is the analysis of the semantic fields used in the discursive strategies due to the impact that hedonistic advertising might have on food disorders, especially in an audience as sensitive to certain stimuli as children. Another relevant aspect is the analysis of the use of celebrity endorsements. Football and basketball players are commonly used to attract consumer attention, an advertising strategy that is usually linked to low nutritional value products and is especially effective among children [54].

Regarding limitations, we should consider that the Nutri-score does not take into consideration ingredients such as caffeine that are highly harmful to children’s consumption. Some products with a medium value are made with caffeine or other ingredients equally harmful to children’s diets.

Although the present study analyzes advertising in different media, as a limitation we must consider that internet advertising is not 100% controlled due to the complexity of the medium itself. Another limitation of this study would be that it focused on only one country. Despite this, the data could be representative of persuasive SSB and SD communications in the Western world.

Finally, a lack of qualitative results based on children’s beverage preferences is another limitation of this research.

## 5. Conclusions

This review indicates that although Spain has a serious problem of childhood obesity, the measures applied are insufficient. Almost all advertising spending on SSBs and SDs goes to products with low or non-existent nutritional values. In addition, the regulatory framework related to the advertising of food and beverages that is applied in Spain has consistent gaps, starting with the fact that the companies supporting the PAOS code, and showing an initial commitment to respecting the regulatory framework contained therein, constantly violate it in their advertising strategies. 

Our results show that, although these companies are constantly launching new healthier beverages to the market, their priority remains the classic sweetened version of their products. This fact has been demonstrated through the advertising analysis classified by the Nutri-score system. In our opinion, a greater involvement of the Spanish beverage industry as well as advertisers and agencies is necessary in order to make the self-regulation of advertising content truly effective in the fight against childhood obesity.

## Figures and Tables

**Table 1:** Nutri-score classification of the advertising spending by the 10 top companies in soft drinks (SDs) and sugar-sweetened beverages (SSBs) in Spain by year.

Year	Total Spending ^1^ *n*	A (%)	B (%)	C (%)	D (%)	E (%)
2013	31,920,827	0.0	13.5	6.9	27.0	52.6
2014	30,911,610	1.6	3.4	6.9	16.4	71.7
2015	40,220,188	0.5	5.1	2.5	25.4	66.5
2016	46,350,870	0.0	6.7	11.1	15.6	66.6
2017	54,262,469	0.1	10.1	16.4	10.9	62.5
2018	53,105,075	0.1	12.0	13.9	16.8	57.2
Total 2013–2018	256,771,039	0.3	8.7	10.4	17.9	62.7

^1^ Spending in EUR.

**Table 2:** Nutri-score classification of the advertising spending by the 10 top companies in soft drinks and sugar-sweetened beverages in Spain by category (2013–2018).

Beverage Type	Total Spending ^1^ *n* (%)	A (%)	B (%)	C (%)	D (%)	E (%)
SDs	218,950,355 (85.3)	0.4	10.2	10.5	14.0	64.9
SSBs	37,820,684 (14.7)	0.0	0.2	9.8	49.4	40.7
Total	256,771,039 (100)	0.0	9.0	10.0	19.0	61.0

^1^ Spending in EUR.

**Table 3:** Nutri-score classification of the advertising spending by the 10 top companies in soft drinks and sugar-sweetened beverages in Spain by media.

Media	Spending ^1^ *n* (%)	A (%)	B (%)	C (%)	D (%)	E (%)
Television	184,536,480 (71.9)	0.4	8.8	11.9	22.0	56.9
Outdoor	38,473,073 (15.0)	0.0	7.4	6.1	12.7	73.8
Cinema	9,428,309 (3.7)	0.0	8.7	5.6	2.5	83.2
Radio	7,216,164 (2.8)	0.0	0.1	13.0	14.3	72.6
Internet	6,404,490 (2.5)	0.5	10.9	11.7	22.8	54.1
Press	5,644,417 (2.2)	0.0	14.0	5.3	7.7	73.0
Magazines	5,072,412 (2.0)	0.1	20.5	1.1	10.5	67.8
Total 2013–2018	256,771,039 (100)	0.3	8.7	10.5	19.2	61.3

^1^ Spending in EUR.

**Table 4:** Nutri-score classification of the advertising keywords by semantic field.

Label	Beverage Qualities	Moods	Action	Leisure Time	Beverages	Meals	Price
	Ads *n* (%)	Ads *n* (%)	Ads *n* (%)	Ads *n* (%)	Ads *n* (%)	Ads *n* (%)	Ads *n* (%)
A	1 (0.8)	1 (3.3)	0 (0.0)	1 (4.5)	0 (0.0)	0 (0.0)	0 (0.0)
B	22 (17.5)	8 (26.7)	7 (24.1)	3 (13.6)	3 (17.6)	2 (28.6)	0 (0.0)
C	20 (15.9)	3 (10.0)	2 (6.9)	4 (18.2)	1 (5.9)	3 (42.9)	0 (0.0)
D	37 (29.4)	8 (26.7)	9 (31.0)	4 (18.2)	8 (47.1)	0 (0.0)	3 (100.0)
E	10 (33.3)	46 (36.5)	11 (37.9)	10 (45.5)	5 (29.4)	2 (28.6)	0 (0.0)
Total ^1^	126 (53.8)	30 (12.8)	29 (12.4)	22 (9.4)	17 (7.3)	7 (3.0)	3 (1.3)

^1^ Total *n* = 234.
